# Placental Sampling for Understanding Viral Infections — A Simplified Protocol for the COVID-19 Pandemic

**DOI:** 10.1055/s-0041-1729146

**Published:** 2021-06-28

**Authors:** Guilherme de Moraes Nobrega, José Paulo Siqueira Guida, Rodolfo Rosa Japecanga, Arthur Antolini-Tavares, Indira Mysorekar, Maria Laura Costa

**Affiliations:** 1 Universidade Estadual de Campinas, Campinas, SP, Brazil; 2 Washington University School of Medicine, St. Louis, MO, United States of America

**Keywords:** COVID-19, placenta, pregnancy, systematic sampling, viral infections, COVID-19, placenta, gestação, coleta sistemática, infecções virais

## Abstract

**Objective** The coronavirus disease 2019 (COVID-19) is a pandemic viral disease, caused by severe acute respiratory syndrome coronavirus 2 (SARS-CoV-2). The impact of the disease among the obstetric population remains unclear, and the study of the placenta can provide valuable information. Adequate sampling of the placental tissue can help characterize the pathways of viral infections.

**Methods** A protocol of placental sampling is proposed, aiming at guaranteeing representativity of the placenta and describing the adequate conservation of samples and their integrity for future analysis. The protocol is presented in its complete and simplified versions, allowing its implementation in different complexity settings.

**Results** Sampling with the minimum possible interval from childbirth is the key for adequate sampling and storage. This protocol has already been implemented during the Zika virus outbreak.

**Conclusion** A protocol for adequate sampling and storage of placental tissue is fundamental for adequate evaluation of viral infections on the placenta. During the COVID-19 pandemic, implementation of this protocol may help to elucidate critical aspects of the SARS-CoV-2 infection.

## Resumo

**Objetivo** A doença do novo coronavírus (COVID-19) é uma doença viral pandêmica causada pelo coronavírus da síndrome respiratória aguda 2 (SARS-CoV-2). O impacto da doença entre a população obstétrica ainda é incerto, e o estudo da placenta pode fornecer informações valiosas. Assim, a coleta adequada do tecido placentário pode ajudar a caracterizar algumas propriedades das infecções virais.

**Métodos** Um protocolo de coleta placentária é proposto, objetivando a garantia de representatividade da placenta, descrevendo a maneira de conservação adequada das amostras, e visando garantir sua integridade para análises futuras. O protocolo é apresentado em suas versões completa e simplificada, permitindo sua implementação em diferentes configurações de infraestrutura.

**Resultados** A amostragem com o intervalo mínimo possível do parto é essencial para coleta e armazenamento adequados. Esse protocolo já foi implementado durante a epidemia de vírus Zika.

**Conclusão** Um protocolo para coleta e armazenamento adequados de tecido placentário é fundamental para a avaliação adequada de infecções virais na placenta. Durante a pandemia de COVID-19, a implementação deste protocolo pode ajudar a elucidar aspectos críticos da infecção por SARS-CoV-2.

## Introduction

Coronavirus disease 2019 (COVID-19) is a severe and highly relevant viral disease in the global scenario. Severe acute respiratory syndrome coronavirus 2 (SARS-CoV-2) (family *Coronaviridae* , genus *Betacoronavirus* ), the etiological agent of the disease, causes asymptomatic or a mild respiratory infection in the majority of cases. 1 2 3 4 However, people with underlying risk factors, such as increased age, cardiovascular disease, and diabetes, present higher rates of clinical complications and severe acute respiratory syndrome (SARS). 4

In viruses of the same genus, such as SARS-CoV (subgenus *Sarbecovirus* ) and Middle East respiratory syndrome coronavirus (MERS-CoV) (subgenus *Merbecovirus* ), as well as in other respiratory disease viruses, there is an increased risk of morbidity and mortality during pregnancy. 3 5 6 7 8 The impact of COVID-19 on the obstetric population and the gestational consequences of SARS-CoV-2 are a great concern for investigation. 9 10 11 Data from the United Kingdom show that most women admitted with SARS-CoV-2 infection during pregnancy were in the late second or third trimester, which replicates the pattern seen for other respiratory viruses, with women in later pregnancy being more severely affected, a third of whom had preexisting comorbidities. 12 In United States, reports show higher rates of hospitalization (31.5%), intensive care unit (ICU) admission (1.5%), and mechanical ventilation (0.5%) in pregnant women, when compared with nonpregnant women (5.8%, 0.9%, 0.3%, respectively). 13 Thus, recent data from Brazil have demonstrated an increased risk of severity among pregnant women, with high numbers of maternal death, and significant cases without adequate respiratory support and with no intensive care admissions. 14

To understand the different facets of COVID-19 during pregnancy, the placenta can serve as a valuable source of information about maternal and fetal conditions. The placenta is a complex and unique interface between maternal and fetal vascular beds, mediating the exchange of nutrients and others residues, allowing the fetal uterine existence and maintaining a highly reliable homeostasis. 15 16 The broad spectrum of placental functions depends on its tissues and cellular stratification, which form a selective biological barrier, called the blood-placental barrier. 17 Those tissues may be affected by viral infections, such as parvovirus B19, rubella virus, cytomegalovirus, herpes simplex viruses, and Zika virus (ZIKV), and the consequences of the placental tissues' immune response and destruction during different periods of pregnancy can lead to severe consequences on gestational and neonatal outcomes. 18

The current evidences about vertical transmission are uncertain and the preponderance of evidence so far does not indicate a significant role for vertical transmission. 5 10 19 However, to understand the impact of COVID-19 on maternal morbidity and mortality is crucial, and the evaluation of the placental tissue may provide data about pathways related to the viral infection within the placenta. 20 Recent placental histopathology results from SARS-CoV-2-positive women did not demonstrate a specific pathology or pathological pattern; however, nonspecific histomorphologic changes suggestive of maternal/fetal vascular malperfusion have been reported. 21 Viral particles in the organ has been detected, although aspects of the effects and pathways of infection by SARS-CoV-2 and how it occurs on placental tissues remain largely unknown up to this date. 21 22 23 24

Our group has previously shown, during the ZIKV outbreak in Brazil, that the placenta is a possible site for viral persistence and that viral detection relies on adequate and appropriate sampling and storage. 25 Thus, here we detail sampling procedures and also propose a simple protocol that can be performed in the delivery room, to guarantee representative tissues of placenta, allowing further investigation consequences of viral infection in pregnancy, including SARS-CoV-2 infection.

## Methods

### Placental Sampling Protocol

**Fig. 1:**
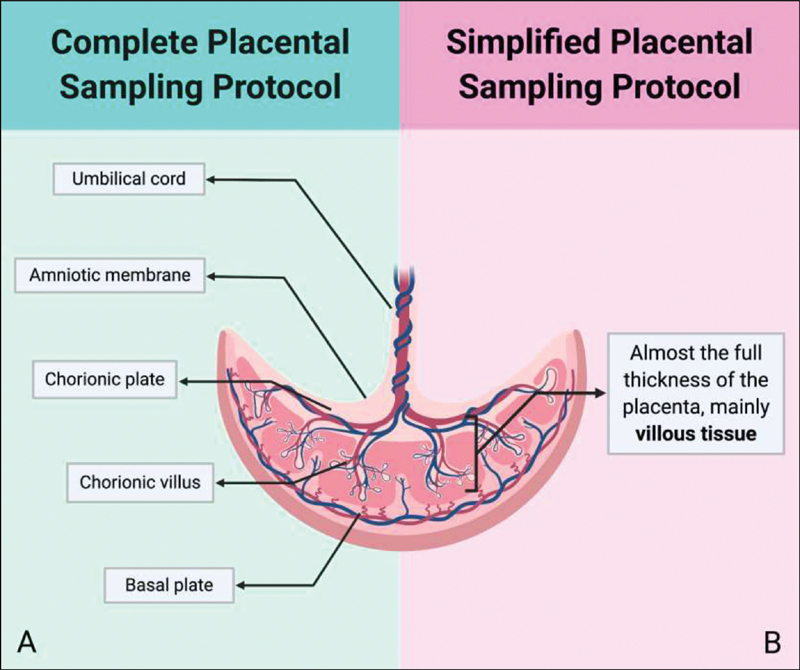
Cross section of the placenta showing its components in different versions of the protocol—complete (A) and simplified (B) placental protocol version.

The placental sampling protocol aims to represent the various tissues that constitute the placenta, and also the umbilical cord, at the time of childbirth. The sampling includes 4 regions of the placenta: the basal plate, the chorionic villus, the chorionic plate, and the amniotic membrane ( Fig. 1 ). To preserve the best sampling quality, collection should be performed in the shortest possible interval from childbirth. Due to different conditions for sampling in different facilities, two versions of the protocol are proposed, the complete ( Fig. 1A ) and the simplified ( Fig. 1B ). All procedures must be performed following the local biosafety rules. The current manuscript is a protocol description. Each study that implements it must necessarily undergo appropriate ethical approval. The protocol was approved by the ethics committee of the coordinating center (#4.047.168) and of each participating center, with implementation in 5 obstetric reference centers of the *Brazilian Network of COVID-19 during Pregnancy* (REBRACO, in the Portuguese acronym) up to now. 26 The latest World Health Organization (WHO) recommendations for sampling COVID-19 patients include a biosafety level 2 (BSL2) facility with all adequate caution. 27 Specific procedures with high viral load, like viral isolation, must be conducted in biosafety level 3 (BSL3) facilities. 28

### Complete Placental Sampling

After childbirth, the placenta should be immediately prepared for sampling or preserved in a cool refrigerator (4°C) in a sterile container for a maximum of 2 hours after childbirth until sampling is possible. The processing of the placenta must be done in an adequate sterile hood; the materials and equipment necessary for adequate placental sampling are described in Supplementary Data S1 (online only). Placental samples will be stored in cryotubes and histology cassettes. All storage materials must be properly identified before the procedure, with patient identification and corresponding placental region.

**Fig. 2:**
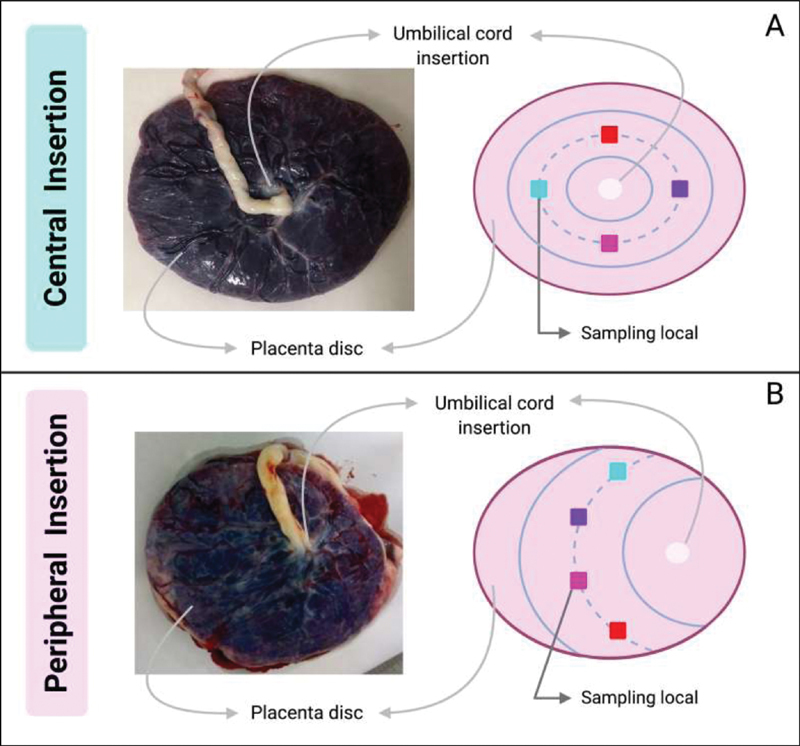
Placental sampling based on umbilical cord insertion site — central (A) and peripheral (B) insertion.

The first step is to have the placenta washed, with sterile saline or sterile phosphate buffer saline, inside a tray, and any solid residues or visible blood clots must be removed. After cleaning, the placenta is placed on a surface with sterile absorbent paper with the basal plate facing up. The choice of the sampling locals to ensure representativeness is based on the insertion of the umbilical cord ( Fig. 2 ). In placentas with umbilical cord centrally inserted, three imaginary concentric circles (one coincident with the placental disc borders, one marginal to the umbilical cord, and a third placed between those two previously described) should be projected, and the sampling places are positioned in the intermediate circle. Four points are chosen in the intermediate circle for sampling, equidistant from each other ( Fig. 2A ). In placentas with peripheral cord insertion, three concentric semi-circles starting from the cord insertion site should be considered, and sampling will be performed in the intermediate circle ( Fig. 2B ). The areas where the sampling take place must not contain macroscopic anomalies, such as areas of detachment or extensive calcification.

After the sampling areas are defined, tissues are sampled in the following order: basal plate, chorionic villus, amniotic membrane, chorionic plate, and umbilical cord ( Fig. 1A ). The basal plate corresponds to the maternal face of the placenta; at the sampling places, an incision should be made with the scalpel 5 mm deep, seeking to avoid contamination with villi. Chorionic villus corresponds to the tissue underlying the basal plate; superficial tissue must be despised because it may contain traces of the basal plate. The amniotic membrane corresponds to the thin and transparent membrane that lines the chorionic plate; to acquire it, this layer must be detached from the chorionic plate. The chorionic plate corresponds to the fetal face of the placenta; it is necessary to dissect the amniotic membrane previously and collect the tissue below, ∼ 2 mm thick, and visible calibrated blood vessels should be avoided. Finally, for the umbilical cord, samples are obtained sectioning it transversely, to obtain two samples.

Samples of ∼15 × 15 × 15 mm (except for the amniotic membrane, where the sample is ∼10 × 5 × 2 mm) are initially obtained, from each placental region, and, further, each of these pieces are divided in 3 equal parts (technical triplicate). Umbilical cord samples ∼10 mm thick and divided in 3 equal parts are also obtained.

**Fig. 3:**
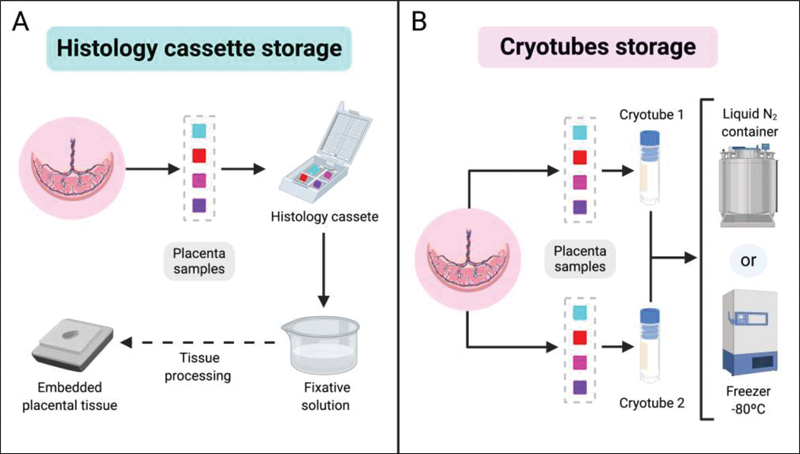
Tissue sample storage process for each sampling site. (A). Histology cassettes storage. (B). Cryotube storage.

Each tissue sampling has two storage destinations: histology cassettes for formalin fixation and cryotubes for cryopreservation. One of three parts of each sample replica will be stored in a cassette, that will in the end contain four samples, one from each previously selected region — again focusing on representativeness ( Fig. 3A ). Two of three parts of each replica will be placed in two cryotubes, each containing 4 samples, one from each previously selected local, guaranteeing the representativeness of the placenta ( Fig. 3B ). The umbilical cord samples follow the same storage method: the histology cassette and each cryotube present two fragments of total section of the tissue.

### Simplified Placental Sampling

After childbirth, the placenta can be immediately sampled in the operating room, by the responsible delivery team, after adequate training. This can mitigate any concerns regarding biosafety standards, materials usage, and adequate use of appropriate personal protective equipment (PPE) ( Supplementary Data 1 ). All storage material must be properly identified with the patient coding, as previously proposed for the complete placental sampling.

The selection of sampling areas to ensure representativeness is the same as that applied for the complete placental sampling, and it is based on the insertion of the umbilical cord, defining four places ( Fig. 2 ). When obtaining such samples, there is no detail on the different regions, and the samples must contain almost the full thickness of the placenta, removing the more superficial maternal tissue and focusing on collecting the villous tissue in deeper regions ( Fig. 1B ). Samples of ∼15 × 15 × 15 mm are obtained from each local.

**Fig. 4:**
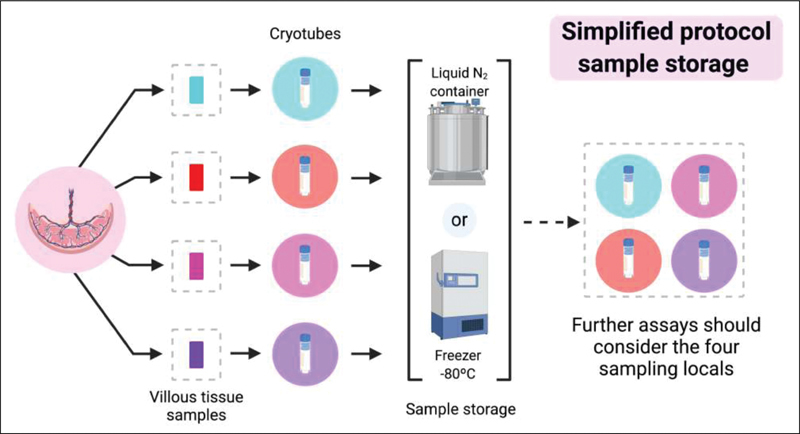
Placenta sample storage process for simplified protocol version.

The samples collected are stored in cryotubes, each containing a sample from a previously selected area, and properly preserved after childbirth. To guarantee representativeness of the placenta, analysis with samples obtained with simplified placental sampling must use material from the four sampling areas for each assay ( Fig. 4 ). After sampling in the operating room, the placenta should be sent for routine pathological analysis.

### Storage and Cautions

Placenta samples in histology cassettes must be placed in a fixative solution, such as 10% buffered formalin, and then be processed for embedding, which can be made in paraffin or other material ( Fig. 3A ). Cryotubes containing samples must be preserved immediately at very low temperatures to maintain sample integrity. Cryotubes must be stored in a liquid nitrogen (N _2_ ) container or directly in -80°C freezer. Low temperatures must be maintained in the final accommodation ( Fig. 3B ). Biosafety guidelines must be followed during all manipulation of samples: sampling, freezing, storing, and processing. 27 28 All disposable materials used during the sampling process must be considered as infectious waste. Other materials must be cleaned and sterilized, preferably in an initial 10% sodium hypochlorite solution. 27

### Histopathological Analysis

The placenta should be further considered for histopathological examination. After the sampling protocol process is finished, the placenta should be placed in a container with an adequate volume of buffered formalin and sent for histopathological analysis. The sampling and analysis of normal and abnormal findings should follow the Amsterdam Placental Workshop Group Consensus Statements, to enable comparison and international standardization of report results. 29 A consistent understanding, with basic gross examination and histologic patterns of injury is important to maximize the diagnostic, prognostic, and therapeutic benefit of placental examination. 30

## Results and Discussion

Adequate placental sampling is key to the evaluation of different insults that may affect the placenta, the woman, and the fetus. We described a placental sampling protocol, which has already been implemented in our setting and allowed us to provide some evidence regarding ZIKV infection during pregnancy. 25 Conserving the integrity of placental samples enables future analysis, using molecular biology and biochemistry techniques.

Immunohistochemistry, immunofluorescence, and a series of different stains, such as the commonly used hematoxylin and eosin stain, can be performed in the histological samples obtained from paraffin-embedded tissue cassettes. Using samples preserved in cryotubes, after specific treatments and extractions, experiments and assays based on proteins (such as Western-blot and proteomic analyzes) or nucleic acids (such as qPCR and next-generation sequencing), or even lipids and other biomolecules, can be implemented. We highlight that some samples are more appropriate than others for specific assays. As a relevant example, formalin-fixated and paraffin-embedded samples could lead to some methodological difficulties for the detection and testing of ribonucleic acid (RNA) viruses, while cryogenic stored samples are more adequate for such experiments.

In addition to maintaining the placental characteristics most similar to those at the moment of childbirth, the sampling is also representative of the placenta as an organ. The samples are collected from areas where the thickness of the placenta is regular, according to its distance from the umbilical cord's spot of insertion. By sampling in random equidistant regions, representativeness of the entire placenta is obtained, thus reducing the interference of specific site features (outliers) and bias in future analyses.

Due to the recommended sampling and subsequent adequate storage, the samples maintain the integrity of the biomolecules. In a previous study from our research group, in which the detailed placental protocol sampling was applied, it was possible to extract whole RNA molecules from samples preserved in -80°C freezers for periods of up to 2 years. This research made it possible to identify the ZIKV genome in placenta samples. This study suggested that, a simplified protocol, mainly with villous tissue samples, if respecting representativeness and adequate storage of the material, could be effective for viral detection. 25 However, inadequate placental sampling can be distracting and generate misleading results; for that reason, all studies involving placental samples should detail the procedure. 31

The infection routes of SARS-CoV-2 regarding vertical transmission remain unclear, and there is limited information about COVID-19 during pregnancy and its consequences. 10 11 32 33 34 Some cellular components have been considered as putative binding receptors for viral entry, such as the membrane protein angiotensin-converting enzyme 2 (ACE2), which is widely expressed in the surface of trophoblasts and endothelial cells. 35 36 37 Recent studies suggest the ACE2 as part of the viral adsorption, and due to its expression in placental cells, it could possibly lead to a placental infection. 38 39 Early studies published had not reported detection of the SARS-CoV-2 genome by reverse transcription-polymerase chain reaction (RT-PCR) assays in placental samples; however, details regarding the methodological process (sampling method, processing time, sample storage) are not clear and, therefore, did not rule out the possibility of viral presence at the maternal-fetal interface, which has now been shown. 40 41 Recent results demonstrated the presence and infection of the virus in the placental tissue, mainly in the chorionic villi, an area emphasized in the current protocol. 22 23 24 A study involving 19 pregnant women infected with SARS-CoV-2 indicated the viral infection in villi syncytiotrophoblast and cytotrophoblasts by *in-situ hybridization* technique (nucleic-acid based technique), with a specific target for the SARS-CoV-2 RNA. 23

As well as the ZIKV, SARS-CoV-2 contains a positive-sense single-stranded RNA genome. 1 3 42 Given the previous experience to detect the ZIKV genome in placentas sampled by this protocol, 25 investigation of the COVID-19 virus could benefit from this protocol. Therefore, the implementation of the simplified protocol focusing on the chorionic villi can enable a greater scope of biological material sampling in different reference centers, mainly in countries with a severe pandemic scenario in the obstetric population, such as Brazil. 14 26

## Conclusion

The placenta has a key role in the understanding of maternal-fetal complications. The implementation of the protocol in different settings would standardize placental sampling and storage, improving techniques description and results, and even providing the exchange of samples through different settings and global locations. The adequate storage of the samples would allow accurate and biologically relevant results in future studies to understand possible critical aspects of viral infections, such as pathogenesis, transmission routes, and functional changes related to infection by SARS-CoV-2 in the placenta.
