# Supplementary material for: Placental Sampling for Understanding Viral Infections — A Simplified Protocol for the COVID-19 Pandemic
Source: Rev Bras Ginecol Obstet. 2021 Jun 28;43(5):377–83. doi: 10.1055/s-0041-1729146 (PMC10305292; doi:10.1055/s-0041-1729146)
Supplement: Supplementary file 1 — Supplementary Material [file 10-1055-s-0041-1729146-s200153.pdf]

Supplementary data 1. Material for placental sampling protocol

Personal Protection Equipment (PPE);
Sterile tray - kidney dish or placenta bucket;
500mL of sterile saline or sterile phosphate buffer saline (not used in simplified version);
5 histology cassettes (not used in simplified version);
Sterile cryotubes (10 vials used in complete version and 4 to 8 vials used in simplified version);
1 sterile scalpel (disposable) or sterile scissors;
1 sterile forceps;
Sterile filter paper or sterile surgery field;
Flask with fixing solution, such as 10% buffered formalin (not used in simplified version);
Liquid nitrogen container or freezer -80°C.

 © 2021. Federação Brasileira de Ginecologia e Obstetrícia. All rights reserved.
